# Meat quality of turkeys after reproductive period depending on genotype and sex^☆^

**DOI:** 10.1016/j.psj.2025.105094

**Published:** 2025-03-24

**Authors:** Marcin Wegner, Dariusz Kokoszyński, Arkadiusz Nędzarek, Joanna Żochowska-Kujawska, Marek Kotowicz, Michał Gesek

**Affiliations:** a Boehringer-Ingelheim, Warsaw, Poland; b Department of Animal Breeding and Nutrition, Faculty of Animal Breeding and Biology, Mazowiecka 28 St., 85-084 Bydgoszcz University of Science and Technology, Bydgoszcz, Poland; c Department of Aquatic Bioengineering and Aquaculture, Faculty of Food Sciences and Fisheries, West Pomeranian University of Technology, Kazimierza Królewicza 4 St., 71-550 Szczecin, Poland; d Department of Meat Science, Faculty of Food Sciences and Fisheries, West Pomeranian University of Technology, Kazimierza Królewicza 4 St., 71-550 Szczecin, Poland; e Department of Pathological Anatomy, Faculty of Veterinary Medicine, University of Warmia and Mazury, Oczapowskiego 13 St., 10-719 Olsztyn, Poland

**Keywords:** Meat quality, Physicochemical trait, Sex, Texture, Turkey parents

## Abstract

With the increasing demand for animal protein, and poultry meat being the most consumed meat in the world, it was decided to perform a genotype- and gender-specific analysis of turkey meat quality. The study was conducted on 48 breast and 48 leg muscles of the two leading genotypes of reproductive turkeys B.U.T. 6 and Hybrid Converter (12 females and 12 males each) after reproductive period. Physicochemical analysis (acidity, electroconductivity, thermal leakage, color L*,a*,b*), basic chemical composition (protein, collagen, water, fat), and micro- and macronutrient content of pectoral muscle and leg muscle were performed. In addition, texture and structure analysis of the pectoralis major muscle was performed. Hybrid Converter's pectoral muscles had significantly (*P* < 0.05) higher fat, water, and iron content compared to B.U.T. 6 pectoral muscles. In comparison, the leg muscles of Hybrid Converter turkeys had a higher content of fat, collagen, sodium and phosphorus, and a lower content of protein, manganese and calcium compared to the analyzed features of the leg muscles of B.U.T. 6. Higher acidity (pH_24_) of pectoral and leg muscles and electro conductivity and horizontal fiber diameter of pectoral muscles were shown in B.U.T. 6 turkeys, while electrical conductivity and yellowness (b*) of leg muscles were higher in Hybrid Converter turkeys. In contrast, the pectoral muscles of males were characterized by significantly (*P* < 0.05) higher pH levels, protein and copper content, and lower water, collagen, potassium, phosphorus, sodium, calcium, thermal loss, and the parameter L*and a*. In contrast, the leg muscles of males were characterized by lower water, collagen, potassium, phosphorus, sodium, calcium, zinc, thermal leakage, and the color parameters a*, b*, and higher fat, chromium, copper, pH, and electrical conductivity. The sex of the birds also affected the texture (Warner-Bratzler shear force, chewiness, gumminess, cohesiveness, sum of elastic moduli) and structure (fiber cross-sectional area, fiber perimeter, vertical fiber diameter, horizontal fiber diameter) characteristics which were higher in males with the exception of sum of elastic moduli. Based on the above information, it can be concluded that the genotype of turkeys largely affects the basic chemical composition, acidity reaction, electrical conductivity, and content of some micro and macro elements of pectoral and leg muscles. The texture, texture, color, and thermal leakage characteristics of the above muscles were not affected by genotype except by the color b* leg muscles and horizontal fiber diameter. On the other hand, a greater effect of gender was observed, especially on the analyzed texture and structure characteristics of the pectoral muscle.

## Introduction

The turkey (*Meleagris gallopavo*) is a bird belonging to the Phasianidae family that was brought to Europe from North America, more specifically from Mexico where the original Guajolote turkeys were domesticated from which the known breeds and varieties are derived (Ribarski and Oblakova, 2016; Portillo-Salgado et al., 2022). World turkey meat production has been steady since 2008 (5.5 - 6.0 million tons) and reached 5.8 million tons in 2021 (Kalman and Szollosi, 2023). The largest producer of turkey meat in 2021 was the US with a share of more than 40 %, followed by Brazil at 9.3 %, while Poland ranked 5th with a share of 5.9 % (Kalman and Szollosi, 2023). Poland is the world's second-largest turkey meat exporting country, with a 17 % share of global exports. Meat consumption worldwide decreased by 0.6 kg per person between 2017 and 2022, while per capita consumption of poultry meat increased by 0.7 kg over the same period (Pardo et al., 2023). The largest consumers of turkey meat per person in 2021 were Israel (9.56 kg/person) and Qatar (6.97 kg/person) (Kalman and Szollosi, 2023). Turkey meat is the second most consumed poultry meat worldwide with average annual consumption ranging from 4.0 kg/person in the European Union (EU) to 7.3 kg/person in the United States (Zampiga et al., 2020).

Consumers are increasingly choosing turkey breast meat for dietary and health-promoting reasons, as it is a rich source of protein, but is also low in fat and cholesterol (Ribarski and Oblakova, 2016). It has a high nutritional value, as it is rich in micronutrients including selenium, magnesium, iron, phosphorus, potassium, and zinc, and also contains the main essential amino acids - lysine, leucine, arginine, and valine (Ribarski and Oblakova, 2016; Zampiga et al., 2020). According to Hristakiev (2006), depending on the genotype and meat type of turkey broilers, the protein content oscillates between 24.72 % - 25.71 %, while the fat content is at 0.31 % - 0.90 %. In contrast, Oblakova et al. (2016) report that the protein content in 20-week-old turkeys was 20.03 % - 23.04 % in breast muscle and 20.73 % - 21.74 % in leg muscle.

From the consumer's point of view, the main determinant of meat choice is its color, and texture, firmness, and water retention capacity are also important aspects (Mir et al., 2017; Leishman et al., 2021; Hiscock et al., 2022). Meat color is influenced by the acidity (pH) of the meat, which, with a sharp drop in pH after slaughtering, causes protein denaturation, which affects water retention capacity, resulting in a lighter meat color (Mir et al., 2017). A large loss of water during thermal processing is undesirable because the meat loses its nutritional value (Abubakar et al., 2021).

Among the most important meat traits that affect culinary and technological value, we can include tenderness, which largely depends on genetic and environmental factors (Rayan et al., 2022). Other authors observed that the hardness of turkey breast muscles depended on muscle activity before slaughter (Leishman et al., 2022). Birds that exercised were characterized by a decrease in pH in the pectoral muscles which was associated with greater thermal leakage, which influenced higher shear force values, which in turn indicate higher meat hardness. Nevertheless, the researchers focus only on analyzing the physicochemical properties of the muscles and the texture, which is mainly based on the shear force value.

There was no information on the texture characteristics (except for WB shear force) and structure of breast muscles as well as an electrical conductivity of aged turkey meat in the available literature. Moreover, there was no comparative study on the quality of meat of B.U.T. 6 turkeys with Hybrid Conventer after the reproductive period, which was the reason for conducting these studies. In this research, the analysis of physicochemical properties, texture characteristics, and pectoral muscle structure of aged parent turkey in relation to genotype and sex was performed. In addition, quantitative measurement of mineral components of the two most popular sets of breeding turkeys in Poland after reproductive period was performed.

## Materials and methods

The study was conducted on pectoral muscles (PMs) and leg muscles (LMs) that came from parent flocks of turkeys after reproductive period at 60 weeks of age. Breast muscles and leg muscles were purchased in May 2023 at the abattoir located in the Warmian-Masurian Voivodeship (north-eastern Poland) from 12 randomly selected females and 12 randomly selected males of B.U.T. 6 and 12 males and 12 females of Hybrid Converter (H.C.). A total of 48 samples of pectoralis major muscle weighing 500 g each and 48 samples of thigh muscles weighing 500 g each were collected and examined. From the breeder's information, during the reproductive period, the birds were kept in turkey houses without windows with regulated environmental conditions. The temperature was 13-14°C and humidity was 65-75 %, females were given a granulated feed mixture containing 11.8 MJ ME and 16.5-18.5 % crude protein (23-45 weeks of age) and 11.7 MJ ME and 15-16.5 % crude protein (46-60 weeks of age), while males were given a granulated feed mixture containing 12 MJ ME and 14-15 % crude protein. After a 12-hour fast, the birds were delivered to the slaughterhouse and were killed at the slaughterhouse in accordance with the guidelines of Council Regulation (EC) No 1099/2009. The birds were transported from the farm to the slaughterhouse in a specialized truck intended for transporting turkeys. The time needed to transport turkeys from the farm to the slaughterhouse was 45 min (B.U.T. 6 turkeys) or 60 min. (H.C. turkeys).

### Physicochemical Properties

After 24 h post-slaughter, the acidity (pH_24_) and electrical conductivity (EC_24_) of the PM (48 pieces) and LM (48 pieces) were analyzed. The pH_24_ was measured using a CX-701 pH meter (Elmetron, Zabrze, Poland), which was calibrated in pH 7.0 and 4.0 buffers. Electrical conductivity was then measured using an LF-Star CPU conductivity probe (Ingenieurburo R. Matthaus, Nobitz, Germany). The electrode was placed at a 90° angle along the muscle fibers of the PM and LM being evaluated. Muscle color was then evaluated using a MINOLTA CR 400 colorimeter (Konica Minolta Chiyoda-ku, Japan, 2013) in the L* (brightness), a* (redness), and b* (yellowness) color system. A 50 mm aperture and a D65 light source were used for the measurement. The colorimeter was calibrated against a white reference plate (*Y* = 94.40, *x* = 0.3159, *y* = 0.3325. Color coordinates were determined in an enclosed air-conditioned room equipped with artificial lighting, with a temperature of 20°C. Three replicates of each sample were performed. For cooking loss analysis, samples weighing 20 ± 2 g were prepared from each of the PM and LM and were wrapped in gauze and placed in an 85°C water bath for 10 min. The samples were then cooled for 30 min at 2°C in a refrigerated cabinet. After cooling, the samples were weighed using an electronic balance, and the weight loss was calculated, which was expressed as a percentage of the initial weight based on the difference in the weight of the sample before and after heat treatment.

### Analysis of basic chemical composition

Analysis of basic chemical composition (water content, protein content, fat content, and collagen content) was performed by near-infrared (NIR) transmission spectroscopy using FoodScan apparatus (FoodScan, Hillerød, Denmark). Ninety grams of meat were taken from each PM and LM sample and ground in an electric meat grinder (Zelmer, Rzeszow, Poland). Four replicates were performed for each sample for chemical composition determination, using the method described by Wegner et al. (2024b).

### Analysis of micro- and macroelements

Elemental composition: Chromium (Cr), Calcium (Ca), Potassium (K), Copper (Cu), Iron (Fe), Mg (Magnesium), Phosphorus (P), Sodium (Na), Manganese (Mn) and Zinc (Zn) were determined in muscles pre-dried (freeze-drying in an Alpa 1-4 LD plus Christ freeze dryer at a temperature of minus 50°C, 0.63 atm, for 24 hrs) to a constant weight and then dissolved by the wet method with a 6 mL mixture of ultrapure concentrated acids HNO3 and HClO4 (Merck, Darmstadt, Germany), mixed in a 5:1 ratio. Muscle samples with an average weight of 0.500±0.001 g were dissolved. The process was carried out using a Speedwave Xpert high-pressure microwave mineralizer, from Berghof (Eningen, Germany). The dissolved samples, after cooling to room temperature, were diluted with Milli-Q water (18.2 MΩ) to a volume of 25 mL. Metals were determined using a ZA300 atomic absorption spectrophotometer from Hitachi (Tokyo, Japan). Calcium, Na, Mg, and K were determined by the acetylene/air flame atomization technique (FAAS) while Cu, Cr, Mn, Zn, and Fe were determined by the graphite cuvette atomization technique in an argon atmosphere (GFAAS). Using a UV-VIS U-2900 spectrophotometer from Hitachi (Tokyo, Japan), the P content was determined using the colorimetric method. A method using ammonium molybdate and ascorbic acid as a reducing agent was used. Absorbance was measured at a wavelength of *l* = 882 nm. In the measurements, calibration curves were prepared based on certified standard solutions from Scharlau (Barcelona, Spain) for Fe, Na, Fe, Mg, K, and Ca and from Merck (Germany) for Cu, P, Zn, Cr, and Mn. To evaluate the accuracy and reliability of the measurements taken, elemental determinations were carried out in Dogfish Liver NCR-DOLT-5 reference material (National Research Council Canada). Elemental recoveries were 107 % (for P), 93 % (for Cr), 98 % (for Zn), 99 % (for Mg), 104 % (for Ca), 103 % (for Fe, Mn), 101 % (for Cu), 114 % (for Na), 98 % (for Zn) and 106 % (for K).

### Texture Analysis

Forty-eight samples of the heat-treated *pectoralis major* muscles taken from 24 B.U.T. 6 and 24 H.C. birds (and in each of the two groups, the research material was collected from 12 females and 12 males) were tested to obtain their textural properties based on texture profile analysis (TPA) as well as Warner-Bratzler (WB) procedure (Bourne, 1982) using a Stable Micro Systems TA.XT Plus (Stable Micro Systems, Godalming, UK). The test involved driving a 0.61 cm diameter shaft twice, parallel to muscle fiber into a sample down to 80 % of their original height (16 mm). A crosshead speed of 50 mm/min-1 and a load cell of 50 N were applied. The force-deformation curves served to calculate muscle hardness, cohesiveness, springiness, chewiness, and gumminess. Shear force determination was conducted on a muscle sample cut to about 10 × 10 × 20 mm using a triangle knife set perpendicular to the muscle fiber. A crosshead speed of 50 mm min^-1^ and a load cell of 500 N were used. The TPA and WB tests were repeated 4 times for each sample.

Rheological properties were determined with the relaxation test run on the Instron 1140 (Intron, Boston, MA, USA). A sample was compressed to 10 % of its original height (2 mm) using a 0.96 cm diameter shaft and left for 90 s. To calculate the elastic and viscous moduli, the general Maxwell's body model was used. The model involves a parallel coupling of a Hookes body and two Maxwell's bodies (Lachowicz, 1992).

The following relaxation equation was applied:

$$\delta =\varepsilon \cdot \left[E_{0}+E_{1}\cdot \exp\left(\frac{-E_{1}\cdot t}{\mu_{1}}\right)+E_{2}\cdot \exp\left(\frac{-E_{2}\cdot t}{\mu_{2}}\right)\right]$$

where: σ, stress; ε, strain; E0, E1, E2, elasticity moduli of Hook's body and of the first and second Maxwell's bodies, respectively; µ1, µ2, viscosity moduli of the first and second Maxwell's bodies, respectively; t, time. Calculated values of the three elastic moduli are summarized as their sum; similarly, the values of the two viscous moduli are presented as their sum. The relaxation test was run 3 times on each muscle.

### Microstructure Analysis

Analysis of pectoral muscle structure was performed on raw meat from 48 carcasses. Three samples of approximately 6 × 6 × 10 mm were taken from each muscle, fixed in Sannomiya solution, dehydrated in alcohol (99.8 %) and benzene and at the end embedded in paraffin blocks. The blocks were cut into 10 µm diameter sections using a microtome, which were placed on slides and stained with contrast (Bruck, 1975). Analytical software and computer image Multi Scan Base v.13 were used to measure fiber cross-sectional area, fiber circumference, horizontal fiber diameter (H), and vertical fiber diameter (V), as well as endomysium thickness and perimysium thickness. Additionally, 10 primary muscle fiber bundles were analyzed per muscle. Furthermore, more than 400 muscle fiber and endomysium and perimysium thickness/samples were analyzed. A magnification of 100 × was applied.

### Statistical Analyses

The numerical data collected in this study were characterized by generally accepted methods of statistical analysis. For each trait studied, the arithmetic means of both factors studied (genotype and gender) and the standard error of the mean (SEM) for both genotypes combined were calculated. Using the Shapiro-Wilk test, the empirical consistency of the trait distribution to a normal distribution was assessed. Therefore, a parametric test in the form of a two-factor analysis of variance was used to determine the effect of genotype and gender on the trait under study. The following linear model was used: Y_ijk_ = µ + a_i_ + b_j_ + (*a* × *b*)_ij_ + e_ijk_ where Y_ijk_ is the trait value, µ is the overall trait mean, ai is the effect of the i th genotype, b_j_ is the effect of the j-th sex, (*a* × *b*)_ij_ is the genotype of the birds by sex interaction, eijk is the error, and k is the kth observation for the target trait in the group.

SAS software (SAS Institute, Cary, NC, USA) version 9.4 was used. The significance of differences at *P* < 0.05 was verified between genotypes and between males and females using Tukey's post hoc test.

## Results

### Chemical Analyses

The genotype of the turkeys had a significant (*P* < 0.05) effect on the percentage of protein and intramuscular fat in PM and LM, and the percentage of water in PM and collagen in LM (Table 1). In contrast, the sex of the birds had a significant effect on water and collagen content in PM and LM, and protein content in PM and intramuscular fat in LM. The pectoral muscles of H.C. turkeys had higher levels of fat and water (*P* < 0.001 - 0.032), while LMs contained more fat and collagen (*P* = 0.006, 0.036; respectively). The LMs of B.U.T. 6 turkeys contained more protein (*P* < 0.001). Males had a higher proportion of protein and lower water in PM (*P* < 0.001), and a higher proportion of fat and lower collagen and water in LM (*P* < 0.001) compared to females. There was also an interaction between genotype and sex in protein and water content in LM (*P* < 0.001, 0.001; respectively).

**Table 1:** Basic chemical composition of breast and leg muscle from 60-week-old commercial parent turkeys.

Trait		Genotype		Sex		SEM	*P -* Values		
		B.U.T. 6	H.C.	Male	Female		G	S	G x S
Protein (%)	PM	23.67^b^	22.90^a^	23.73	22.84*	0.098	<0.001	<0.001	0.925
	LM	20.30^a^	18.64^b^	19.39	19.55	0.140	<0.001	0.147	<0.001
Intramuscular fat (%)	PM	3.39^b^	3.86^a^	3.68	3.57	0.108	0.032	0.589	0.641
	LM	4.67^b^	5.41^a^	6.62	3.46*	0.267	0.006	<0.001	0.517
Collagen (%)	PM	1.53	1.56	1.48	1.61*	0.017	0.330	<0.001	0.153
	LM	1.58^b^	1.66^a^	1.52	1.72*	0.027	0.036	<0.001	0.219
Water (%)	PM	71.05^b^	72.12^a^	70.75	72.42*	0.195	0.001	<0.001	0.735
	LM	70.73	71.02	69.48	72.27*	0.239	0.186	<0.001	0.001

Note: For all chemical traits from each genotype 24 birds were evaluated, in total 48 birds were evaluated, 24 males and 24 females.

^a,b^ Values within a row with different superscripts differ significantly between genotypes at *P* < 0.05.

*statistical differences between males and females at *P* < 0.05.

S - sex; G - genotype; *G* × *S* – interaction between genotype and sex; SEM - standard error of the mean; PM - pectoral muscle; LM - leg muscle; H.C. - Hybrid Converter.

Analyzing the content of macro and micro elements (Table 2) in PM and LM showed a significant (*P* < 0.05) effect of genotype on the content of P, Na, Ca, Mn in LM and Fe in PM. In contrast, the sex of the turkeys significantly affected the content of K, P, Na, Ca, Cu in PM and LM, and the content of Zn and Cr in LM. H.C. leg muscles had higher phosphorus and sodium contents (*P* = 0.009, 0.017; respectively), while calcium and manganese contents were lower (*P* = 0.021, 0.039; respectively). The pectoral muscles of B.U.T. 6 contained more iron (*P* = 0.001). PMs of females were characterized by higher contents of K, P, Na, Ca, and lower Cu (*P* < 0.001 - 0.037). LMs of females were characterized by significantly higher K, P, Na, Ca, Zn, and lower Cu and Cr content compared to males (*P* < 0.001 - 0.022). There was also an interaction between genotype and sex in the analyzed contents of P and Mg in LM, and Mg and Zn in PM (*P* = 0.006 - 0.049).

**Table 2:** Content of selected macro- and micronutrients in meat from 60-week-old commercial parent turkeys.

Trait		Genotype			Sex		SEM	*P-* Values		
		B.U.T. 6		H.C.	Male	Female		G	S	G x S
Macroelements (mg/100 g of meat)										
K - potasium	PM		306.73	306.51	293.96	319.28*	4.901	0.985	0.037	0.484
	LM		386.81	370.01	366.48	390.34*	5.260	0.913	0.001	0.518
P - phosphorus	PM	241.36		239.90	228.43	252.83*	3.260	0.834	0.001	0.356
	LM	242.99^b^		260.38^a^	241.07	262.30*	3.414	0.009	0.002	0.006
Na - sodium	PM	62.89		69.11	51.61	80.39*	3.023	0.263	<0.001	0.820
	LM	84.03^b^		93.73^a^	79.53	98.23*	2.160	0.017	<0.001	0.296
Mg- magnesium	PM	24.48		24.86	24.21	25.13	0.489	0.736	0.427	0.026
	LM	24.80		25.12	24.60	25.31	0.318	0.671	0.356	0.049
Ca - calcium	PM	2.37		1.91	1.52	2.76*	0.138	0.065	<0.001	0.244
	LM	2.89^a^		2.25^b^	2.16	2.98*	0.127	0.021	0.004	0.719
Microelements (mg/100 g of meat)										
Fe - iron	PM	0.41^a^		0.31^b^	0.35	0.37	0.011	0.001	0.582	0.841
	LM	1.70		1.80	1.63	1.87	0.051	0.494	0.068	0.960
Zn - zinc	PM	1.18		1.25	1.23	1.20	0.059	0.600	0.773	0.038
	LM	4.98		5.06	4.63	5.41*	0.137	0.846	0.022	0.723
Cu - copper	PM	0.024		0.022	0.029	0.017*	0.001	0.536	0.001	0.121
	LM	0.112		0.113	0.124	0.101*	0.003	0.889	0.001	0.331
Mn -manganese	PM	0.013		0.022	0.014	0.021	0.003	0.248	0.444	0.481
	LM	0.017^a^		0.005^b^	0.010	0.012	0.002	0.039	0.539	0559
Cr - chrome	PM	0.001		0.001	0.001	0.001	0.001	0.131	0.130	0.843
	LM	0.008		0.007	0.012	0.003*	0.001	0.670	<0.001	0.195

Note: For macro- and microelements in total 32 birds were evaluated, 16 males and 16 females from each genotype.

a,b Values within a row with different superscripts differ significantly between genotypes at *P* < 0.05.

*statistical differences between males and females at *P* < 0.05.

S - sex; G - genotype; *G* × *S* – interaction between genotype and sex; SEM - standard error of the mean; PM - pectoral muscle; LM - leg muscle; H.C. - Hybrid Converter.

### Physicochemical Properties

Analyzing the physicochemical properties of PM and LM of post-reproductive turkeys showed a significant (*P* < 0.05) effect of genotype on pH_24_ and EC_24_ of PM and LM (Table 3). In contrast, the sex of the birds had a significant effect on the pH and thermal loss of PM and LM and EC of LM. PM and LM of B.U.T. 6 turkeys were characterized by higher pH levels (*P* < 0.001). The electrical conductivity of PM of B.U.T. 6 turkeys was higher, while EC of leg muscles was lower than EC of H.C. (*P* < 0.004 - 0.015). Analyzing the thermal leakage value in PM and LM showed no significant differences between genotypes (*P* = 0.311, 0.459; respectively). Males, on the other hand, were characterized by higher pH levels in PM and LM and higher EC_24_ in LM compared to the analyzed traits in females (*P* < 0.001). Females, on the other hand, were characterized by higher thermal leakage in PM and LM compared to males (*P* < 0.001, 0.001; respectively). There was also an interaction between genotype and sex in pH_24_ and thermal leakage in PM (*P* = 0.001, 0.017; respectively).

**Table 3:** Some physicochemical properties of breast and leg muscle from 60-week-old of parent turkeys.

Trait		Genotype		Sex		SEM	*P* - Values		
		B.U.T. 6	H.C.	Male	Female		G	S	G x S
pH_24_	PM	6.47^a^	6.00^b^	6.36	6.11*	0.043	<0.001	<0.001	0.001
	LM	6.57^a^	6.07^b^	6.48	6.16*	0.047	<0.001	<0.001	0.193
EC_24_ (mS/cm)	PM	11.69^a^	10.70^b^	10.84	11.55	0.221	0.015	0.073	0.067
	LM	7.53^b^	9.03^a^	6.97	5.59*	0.326	0.004	<0.001	0.809
Thermal loss (%)	PM	24.98	26.31	23.28	28.01*	0.669	0.311	<0.001	0.017
	LM	32.80	31.78	29.63	34.95*	0.776	0.459	0.001	0.989

Note: For all physicochemical traits in total 48 birds were evaluated, 12 males and 12 females from each genotype.

^a,b^ Values within a row with different superscripts differ significantly between genotypes at *P* < 0.05.

*statistical differences between males and females at *P* < 0.05.

S - sex; G - genotype; *G* × *S* – interaction between genotype and sex, SEM-standard error of the mean; PM - pectoral muscle; LM - leg muscle; H.C. - Hybrid Converter.

Analyzing the effect of genotype on L*, a*, b* levels of pectoral and leg muscles showed no significant (*P* < 0.05) effect, except for the level of b* color saturation in LM (Table 4). The LMs of H.C. turkeys were characterized by higher levels of b* color relative to the trait analyzed in B.U.T. 6 birds (*P* < 0.001). On the other hand, significant differences in PM and LM color were shown according to sex. The pectoral muscles of females were characterized by higher levels of L* and a* color saturation, while the leg muscles were characterized by higher levels of a* and b* color (*P* = 0.001 - 0.009). It was shown, also, an interaction between genotype and gender in the level of yellow color saturation in leg muscle and red color saturation in pectoral muscle (*P* = 0.001, 0.041; respectively).

**Table 4:** Color parameters of breast and leg muscle from 60-week-old parent turkeys.

Trait		Genotype		Sex		SEM	*P* - Values		
		B.U.T. 6	H.C.	Male	Female		G	S	G x S
L* − lightness	PM	50.26	49.26	48.44	51.08*	0.403	0.166	0.001	0.718
	LM	37.41	38.67	37.11	38.97	0.576	0.272	0.110	0.605
a* − redness	PM	3.97	4.38	3.69	4.67*	0.197	0.260	0.009	0.041
	LM	14.50	14.02	13.06	15.46*	0.452	0.570	0.007	0.359
b* - yellowness	PM	1.19	3.73	3.59	1.33	0.338	0.349	0.402	0.255
	LM	1.79^b^	4.00^a^	1.87	3.92*	0.358	<0.001	0.001	0.001

Note: For color traits in total 48 birds were evaluated, 12 males and 12 females from each genotype.

a,b Values within a row with different superscripts differ significantly between genotypes at *P* < 0.05.

*statistical differences between males and females at *P* < 0.05.

S - sex; G - genotype; *G* × *S* – interaction between genotype and sex, SEM-standard error of the mean; PM - pectoral muscle; LM - leg muscle; H.C. - Hybrid Converter.

### Texture Analysis

Table 5 shows the texture characteristics of the superficial pectoral muscle and rheological properties. The genotype of birds had no significant (*P* < 0.05) effect on the analyzed texture traits (hardness, WB shear force, springiness, chewiness, gumminess, cohesiveness) and rheological properties (sum of elastic moduli, sum of viscous moduli) (*P* = 0.243 - 0.973). On the other hand, there were significant differences in the analyzed characteristics (WB shear force, chewiness, gumminess, cohesiveness) and the sum of elastic moduli according to the sex of the birds. The pectoral muscles of females were characterized by lower values of the above traits and significantly higher sum of elastic moduli (*P* < 0.001 - 0.016). There was also an interaction between genotype and sex in cohesiveness (*P* = 0.016).

**Table 5:** Texture and rheological parameters of m. pectoralis major in 60-week-old parent turkeys.

Trait	Genotype		Sex		SEM	*P* - Values		
	B.U.T. 6	H.C.	Male	Female		G	S	G x S
Hardness (N)	28.15	28.64	28.11	28.47	0.453	0.589	0.866	0.811
WB shear force (N)	41.72	42.47	47.08	37.11*	1.085	0.719	<0.001	0.455
Springiness (cm)	1.60	1.58	1.59	1.59	0.011	0.506	0.785	0.266
Chewiness (*N* × cm)	17.58	17.60	18.85	16.33*	0.416	0.973	0.002	0.283
Gumminess (N)	11.04	11.87*	11.92	11.31*	0.290	0.789	0.005	0.176
Cohesiveness	0.39	0.39	0.42	0.36*	0.006	0.934	<0.001	0.016
EMS (kPa)	332.97	356.49*	319.86	369.60*	10.41	0.243	0.016	0.945
VMS (Kpa × *s*)	10490	12017	11474	11041	637.86	0.243	0.734	0.353

Note: For all texture and rheological traits in total 48 birds were evaluated, 12 males and 12 females from each genotype.

*statistical differences between males and females at *P* < 0.05.

S - sex; G - genotype; *G* × *S* – interaction between genotype and sex, SEM, standard error of the mean, EMS - sum of elastic moduli, VMS - sum of viscous moduli; H.C., Hybrid Converter.

### Structure Analysis

Analysis of pectoral muscle structure showed the effect of genotype on horizontal fiber diameter and the effect of sex on fiber cross-sectional area, fiber perimeter, vertical fiber diameter, and horizontal fiber diameter (Table 6). The pectoral muscles of H.C. turkeys were characterized by a significantly lower horizontal fiber diameter (*P* = 0.004). In males, on the other hand, higher fiber cross-sectional area, fiber perimeter, vertical fiber diameter, and horizontal fiber diameter were observed compared to the analyzed traits in females (*P* < 0.001 - 0.008). There was also an interaction between genotype and sex in the analyzed structure traits: fiber cross-sectional area, fiber perimeter, vertical fiber diameter, horizontal fiber diameter, endomysium thickness, and perimysium thickness (*P* < 0.001).

**Table 6:** Structure parameters of m. pectoralis major in 60-week-old parent turkeys.

Trait	Genotype		Sex		SEM	*P* Values		
	B.U.T. 6	H.C.	Male	Female		G	S	G x S
Fibre cross-sectional area (µm^2^)	2774.80	2712.00	2996.1	2491.60*	46.19	0.636	<0.001	<0.001
Fibre perimeter (µm)	223.68	217.01	230.43	210.26*	36.10	0.171	<0.001	<0.001
Vertical fiber diameter(µm)	63.05	64.98	67.92	60.11*	1.263	0.274	<0.001	<0.001
Horizontal fiber diameter (µm)	61.32^a^	56.35^b^	61.10	56.57*	1.140	0.004	0.008	<0.001
Endomysium thickness (µm)	2.12	2.04	2.12	2.04	0.042	0.206	0.232	<0.001
Perimysium thickness (µm)	20.33	19.91	19.26	20.97	0.597	0.686	0.107	<0.001

Note: For all structure traits in total 48 birds were evaluated, 12 males and 12 females from each genotype.

^a,b^ Values within a row with different superscripts differ significantly between genotypes at *P* < 0.05.

*statistical differences between males and females at *P* < 0.05.

S - sex; G - genotype; *G* × *S* – interaction between genotype and sex; SEM - standard error of the mean; H.C. - Hybrid Converter.

## Discussion

One of the key factors that determine the quality of meat is the acidity (pH). High meat pH has a very negative effect, as it creates an environment conducive to bacterial growth, which reduces shelf life (Wegner et al., 2024a). In our study, the level of acidity was influenced by the genotype and sex of the birds. The higher pH_24_ in the breast and leg muscles of males in our study was most likely due to the greater muscle mass, i.e. higher glycogen content in the muscles, which has a significant impact on the final pH of the meat. Higher pH_24_ values ​​in the breast and leg muscles of B.U.T. 6 turkeys than in H.C. turkeys were associated with a lower glycogen content (from which lactic acid is produced, causing meat acidification) in the meat of B.U.T. 6 turkeys as a result of the impact of pre-slaughter factors. The results obtained in the pectoral muscle (6.47) and leg muscle (6.57) in the presented study in B.U.T. 6 turkeys were higher compared to those of hybrid turkeys breeder (pH = 5.69, 5.91; respectively) (Chartrin et al., 2019). Leishman et al. (2022) also showed the influence of genotype on the pH value of pectoral muscles, but the results obtained were very similar to those presented in the above study. Turkeys from the line characterized by higher body weight were characterized by significantly higher pectoral muscle pH (Leishman et al., 2022). In another study, however, no effect of the genotype of the turkeys studied on the pH value of the breast muscles was demonstrated, but the effect of the time (1-7 days) of meat storage (breast muscles) on the pH level was demonstrated, the value of which dropped significantly on the 3rd day (Zampiga et al., 2019). Rayan et al. (2022) observed that the acidity reaction is strongly correlated with thermal drip loss and meat color. A rapid drop in pH after slaughter affects the denaturation of muscle proteins, which in turn causes a lower ability of the muscle to retain water and affects the color of the meat, which is with lighter and more yellow (Hiscock et al., 2022).

Meat color largely influences consumer purchasing decisions. In our study, the genotype of birds did not affect the color of the breast and leg muscles, apart from the yellowness of the leg muscles. Most likely, the higher values ​​of b* color saturation in H.C. turkeys were influenced by the higher content of intramuscular fat in the analyzed muscle. In the study by Zampiga et al. (2019), no effect of genotype on the color of the breast muscles was demonstrated, apart from the saturation of the yellow color, which was confirmed in the presented study. Other studies also demonstrated the effect of the genotype of turkeys on the level of saturation of the breast muscles with yellow color (Tavaniello et al., 2019; Leishman et al., 2022). Female lines selected for meat traits were characterized by a higher saturation of the yellow color of the breast muscles compared to the female line selected for reproductive traits or slow-growing turkey lines (Leishman et al., 2022). Chartrin et al. (2019) observed that breast muscles from parent flocks were characterized by a higher level of saturation (L*, a*) compared to turkey broilers and that breast muscles of females were characterized by a higher level of yellow color saturation compared to males. Also in the studies of Portillo-Salgado et al. (2022), the authors showed that with the age of turkeys (24, 32, 40 weeks), the level of saturation of breast and leg muscles with L* and b* decreased, while the level of saturation with a* increased. Our studies also showed the influence of gender on the level of color saturation (L*and a*) of breast muscles, and the level of color saturation (a* and b*) of leg muscles. The breast muscles of females were characterized by a higher level of light color and lower red, while the leg muscles had a higher level of a* and b* color saturation compared to the analyzed traits in males. The lower L* and higher a* values ​​in male breast muscles were most likely influenced by different muscle fiber metabolism, which is influenced by sex hormones (Sirri et al., 2009). Also, the number of red blood cells in males is higher, and consequently, the amount of hemoglobin that affects muscle color is higher. Also in another study, the breast and leg muscles of females were characterized by a lower a* color saturation compared to males (Galvez et al., 2018).

One of the most important components that determines the juiciness of meat after cooking is water content (Rayan et al., 2022). Meat that is characterized by high thermal leakage is not very attractive to consumers because it loses valuable nutrients during cooking and is dry (Wegner et al., 2023). Leishman et al. (2022) and Rayan et al. (2022) analyzed the thermal leakage of the breast and leg muscles and did not show any significant differences between the genotypes in the analyzed trait, which was confirmed in the presented study. The authors Hiscock et al. (2022) observed, however, that female lines were characterized by lower thermal leakage of the breast muscle compared to the analyzed trait in the male line (selected for meat traits). Also in our study, the breast and leg muscles of females were characterized by higher thermal leakage compared to the analyzed trait in males. The results obtained in our study were most likely influenced by the higher acidity level in the breast and leg muscles in males, which resulted in a lower level of thermal leakage. Also Portillo-Salgado et al. (2022) confirmed the influence of turkey sex on the level of thermal leakage in the breast muscle. The same authors also observed that the age of the birds had a significant influence on the above trait. The breast muscles of turkeys at 32 weeks of age were characterized by the highest thermal leakage compared to the analyzed trait of birds slaughtered at 24 and 40 weeks (Portillo-Salgado et al., 2022). Chartrin et al. (2019) also confirmed in previous studies that breast muscles from parent flocks and males were characterized by higher thermal leakage compared to young birds and females. Thermal drip is strongly correlated with meat pH, which is influenced by many factors. With age, meat pH decreases, which has a key impact on thermal drip, which is greater. Older birds are also more fatty, which affects greater meat weight losses during cooking.

Another trait that is closely related to thermal leakage and water absorption, and which determines the quality of meat is EC. A large decrease in EC within 24 h after slaughter may indicate higher thermal leakage and lower water absorption of meat (Wegner et al., 2024b). According to Turgay-Izzetoglu et al. (2022), the EC of meat is primarily influenced by its composition, in particular by its fat content and the direction of muscle fibers. In our study, the breast and leg muscles of H.C. turkeys were characterized by higher EC values ​​compared to the analyzed trait in B.U.T. 6. Most likely, the results obtained were influenced by the higher intramuscular fat content in H.C. turkeys. In the present study, the genotype and sex of birds also influenced the content of protein, water, and collagen in the breast and leg muscles. Higher protein content and lower fat content were observed in the breast and leg muscles of B.U.T. 6 turkeys. On the other hand, males were characterized by higher protein levels in the breast muscle and lower collagen and water levels in the breast and leg muscles. In other studies, it was shown that females were characterized by higher levels of protein and dry mass in the breast and leg muscles compared to the analyzed traits in males (Portillo-Salgado et al., 2022). Chartrin et al. (2019) also observed higher protein levels in the breast muscle in females and showed that the protein level in the breast muscle decreased significantly with the age of the birds.

The present study showed that H.C. turkeys after the reproductive period were characterized by higher levels of potassium and magnesium, lower manganese in the leg muscle, and lower iron levels in the breast muscle compared to the analyzed traits in B.U.T. 6 turkeys. Females, on the other hand, were characterized by higher content of macro (K, P, Na and Ca) and lower micro (Zn and Cu) elements in the breast and leg muscles. In our study, it was observed that the breast and leg muscles contained the most potassium and phosphorus, which was also shown in other studies (Chen et al., 2016; Pieterse et al., 2019). On the other hand, the authors Ribarski and Oblakova (2016) observed that the breast muscles of free-living turkeys contained the most Fe - 1.34 %, and the least Mg - 0.02 %, while the share of potassium was at the level of 0.37 %, and phosphorus - 0.26 %. Williams et al. (2017) analyzed the content of some micro and macro elements in the breast muscle of broiler turkeys and showed similar values ​​of magnesium, while the value of phosphorus (180 mg/100 g) and potassium (229 mg/100 g) were much lower compared to the results in the presented study (P – 240 mg/100 g; K – 306 mg/100 g). However, similar values ​​of P, and K in the breast muscle and leg muscle were observed in another study (Galvez et al., 2018). The same authors showed, however, that the breast muscles of males were characterized by a significantly higher level of Na, and the leg muscles by a higher level of Fe, Na, and Zn compared to the analyzed features in females. Ribarski and Oblakova (2016) did not show any differences in the content of minerals in the breast muscle depending on sex in turkeys living in the natural environment. In our study, we confirmed previous reports (Zampiga et al., 2019; Hiscock et al., 2022; Leishman et al., 2022) that the genotype of birds does not affect the WB value of the shear force of the breast muscle in turkeys. This study also showed that the genotype of turkeys did not affect the hardness, springiness, chewiness, gumminess, cohesiveness, and rheological properties (sum of elastic moduli, sum of viscous moduli). The higher values ​​of the texture features of the pectoral muscle in males compared to females were due to the structure of the muscle fibers. The pectoral muscles of males are characterized by a larger cross-sectional area, fiber perimeter, vertical and horizontal fiber diameter than females, which affects the texture features of the muscle. On the other hand, Werner et al. (2008) showed that the breast muscles of Kelly Wrolstad turkeys between 42 and 46 weeks of age were characterized by the lowest significant WB value of the shear force compared to other genotypes tested (B.U.T. Big 6, Kelly BBB, Kelly Super Mini). In the present study, it was shown that the male breast muscles were significantly more cohesive, rubbery, hard, and chewy, which confirmed previous findings (Chartrin et al., 2019). Leishamn et al. (2022) did not show significant differences in the WB shear force of the breast muscle between 6 different genotypes, but they did show differences depending on the season. In summer, the WB value of the shear force of the breast muscles in turkeys was higher than in autumn.

In the presented study, it was observed that the genotype of turkeys influenced the value of the horizontal fiber diameter. Also in the study by Werner et al. (2008), the authors demonstrated the effect of genotype on the fiber diameter. The smallest fiber diameter was characterized by the breast muscles of Kelly Super Mini and Kelly Wrolstad turkeys, while the largest was in Kelly BBB and B.U.T. Big 6. In the study by Chartrin et al. (2019), the authors demonstrated the effect of age and sex on the cross-sectional area of ​​the fiber in hybrid turkeys. The breast muscles of the parent flock and males had a significantly larger cross-sectional area, while the smaller ones were in turkey broilers and females. The results obtained by the authors were very similar to the results in our study of birds of the same genotype and age. The presented study also confirmed that the breast muscles of males were characterized by a higher fiber cross-sectional area, fiber perimeter, vertical fiber diameter, and horizontal fiber diameter compared to females. The authors Okuskhanova et al. (2017) showed the fiber diameter in turkeys (broiler) at the level of 37.71 µm to 60.93 µm, which was lower compared to the results in our study (H.C. – 56.35 µm, B.U.T. 6 – 61.32 µm).

In conclusion, the breast and leg muscles of B.U.T. 6 turkeys are characterized by higher protein content, lower fat content, and higher acidity. The genotype of turkeys did not affect the analyzed features of thermal drip, texture, structure, and color, except for horizontal fiber diameter and b⃰ color of leg muscles. However, from the point of view of sex, females are characterized by more favorable properties of texture, structure, and content of macroelements. The provided results can support the processing industry and consumers guided by quality to be able to use turkey meat after the reproductive period.

## Declaration of competing interest

The authors hereby confirm that the manuscript has not been published in whole or in part, nor is being considered for publication elsewhere and all authors are in agreement with the content of the manuscript. The authors declare any financial support from or relationships that may pose conflict of interest.
